# Risk factors associated with long-term shedding infections of non-typhoidal *Salmonella* in humans

**DOI:** 10.1007/s10096-025-05165-x

**Published:** 2025-05-27

**Authors:** Andreas Rohringer, Lamprini Veneti, Anke Stüken, Elburg van Boetzelaer, Hilde M. Lund, Zuzana Nordeng, Emily MacDonald, Umaer Naseer

**Affiliations:** 1 Norwegian Institute of Public Health (NIPH), Oslo, Norway; 2 European Programme for Public Health Microbiology (EUPHEM), European Centre for Disease Prevention and Control (ECDC), Stockholm, Sweden; 3 European Programme for Field Epidemiology (EPIET), European Centre for Disease Prevention and Control (ECDC), Stockholm, Sweden

**Keywords:** *Salmonella*, Non-typhoidal *Salmonella*, Long-term shedding, Prolonged convalescent excretion, Risk factors, Norway

## Abstract

**Purpose:**

Non-typhoidal *Salmonella* (NT*S*) gastroenteritis in humans is typically self-limited, resolving within 48–72 h. However, some infections result in a carrier state characterised by persistent gut colonisation and long-term shedding (LTS). This study aimed to investigate risk factors associated with LTS of NTS in humans.

**Methods:**

Salmonellosis cases reported to the Norwegian surveillance system in 2019 were invited to participate. Participants submitted a follow-up stool sample and a questionnaire five weeks after initial sampling (detecting infection). Stool samples were cultured, and isolates were sequenced to determine genotype, serotype and antimicrobial resistance genotype. NTS cases were classified as LTS if the isolates from both samples differed by ≤ 5 alleles. Adjusted odds ratios (aORs) with 95% confidence intervals (95%CIs) were calculated using logistic regression to investigate potential risk factors associated with LTS.

**Results:**

Of 1,094 reported cases, 255 (23%) with NTS participated; 24% were classified as LTS. Children aged 0–5 years were 6.7 times more likely to exhibit LTS compared to adults aged 18–44 years (aOR = 6.71, 95%CI:1.67–26.94). Participants who received regular medication and those following a lactose-free diet were 2.2 (aOR = 2.17, 95%CI:1.02–4.64) and 7.2 (aOR = 7.24, 95%CI:1.48–35.40) times more likely to exhibit LTS than those who did not, respectively. Participants with S. Agbeni or S. Bron were 6 times more likely to exhibit LTS compared to S. Typhimurium cases (aOR = 6.29, 95%CI:1.40–28.16).

**Conclusions:**

Observed risk factors associated with LTS included young age, regular medication use, lactose-free diet, and specific *Salmonella* serotypes. Further research is needed to increase knowledge regarding LTS and inform infection control measures.

**Supplementary Information:**

The online version contains supplementary material available at 10.1007/s10096-025-05165-x.

## Introduction

Non-typhoid *Salmonella* (NTS) is a Gram-negative, rod-shaped bacteria of the species *Salmonella enterica (S. enterica) that causes* gastrointestinal illness, excluding the serovar Typhi and Paratyphi [1]. In humans, they are generally transmitted through the faecal-oral route, usually through the improper handling or contamination of foods, and zoonotically from infected animals [1–3]. Globally, non-typhoidal *Salmonella enterica* is estimated to cause 79 million infections and about 60,000 deaths which correspond to 4 million disability-adjusted life years annually [4]. In Europe, around 60,000 *Salmonella* infections were reported in 2021, corresponding to a notification rate of 15.7 per 100,000 and showing a stable trend over 2017–2021 [3]. However, not all enteric infections with NTS lead to severe diarrhoea. Instead, they may be clinically mild or even asymptomatic. The severity of the disease depends on the pathogenicity of the *Salmonella* strain as well as host-specific factors like gastric hypoacidity, recent use of antibiotics, age, and a variety of immunosuppressive conditions [5–7].

Symptomatic NTS gastroenteritis in humans is usually self-limiting with fever generally resolving within 48 to 72 h and diarrhoea within 4 to 10 days. Antibiotic therapy for symptomatic diarrheal illness does not consistently prevent or shorten the duration of carriage. On the contrary, some studies show that it is associated with a more extended period of excretion in infected individuals, potentially exacerbating its transmission risk [8, 9]. Antibiotics have also been shown to lead to a “super shedder” phenotype in animals, where *Salmonella* is excreted at very high levels and can spread rapidly in animal populations [10]. The gut microbiota has been suggested to play a critical role in the clearance of *Salmonella*, and its disruption can induce long-term asymptomatic shedding [11].

In both symptomatic and asymptomatic cases, the initial infection can result in a carrier state in humans and livestock, marked by persistent colonisation of the gut and continuous long-term shedding of *Salmonella* [12, 13]. This long-term shedding of Salmonella in faeces—which may continue for weeks or months after infection—may vary in duration depending on the serotype and is often longer following symptomatic infection [12, 13]. Chronic carriage of NTS is defined as the shedding of a *Salmonella* species for more than one year. The median duration of excretion following infection is approximately five weeks. Age younger than five years is associated with a more extended period of excretion, with a median of roughly seven weeks [14, 15]. Among this age group, 2.6% excrete *Salmonella* for more than one year, compared with less than 1% among all age groups [15].

Many pathogen and patient risk factors have been suggested to be associated with the long-term *Salmonella* shedding, such as virulence genes, antimicrobial resistant strains, age, diet, and medication, including antibiotic usage [10, 12, 14–16]. However, no clear evidence has emerged for human long-term *Salmonella* shedding, and more work is needed to explore this phenomenon. To increase knowledge on risk factors associated with human long-term *Salmonella* shedding and potentially inform evidence-based infection control and preventive care measures, we conducted a prospective cohort study investigating host and pathogen-specific risk factors associated with long-term shedding of NTS among salmonellosis notified cases in Norway in 2019.

## Materials and methods

### Set up and study design

In Norway, salmonellosis is a mandatory notifiable disease to the Norwegian Surveillance System for Communicable Diseases (MSIS) located at the Norwegian Institute of Public Health (NIPH). For all cases reported, isolates are sent to the National Reference Laboratory (NRL) for Enteropathogenic bacteria at NIPH, for confirmation and analyses to characterise the *Salmonella* strains.

We conducted a prospective cohort study among non-typhoidal salmonellosis cases reported to MSIS between 1 January 2019 and 31 December 2019. The study was conducted as part of the European “MoMIR-PPC project: Monitoring the gut microbiota and immune response to predict, prevent and control zoonoses in humans and livestock in order to minimise the use of antimicrobials”. The study received funding from the European Union’s Horizon 2020 Research and Innovation program.

### Data collection and recruitment

An invitation to participate in the Norwegian MoMIR study was sent out by post to all salmonellosis cases as soon as they were notified to MSIS during the study period. After consenting, a study package was sent to the participants including a questionnaire, a stool sampling kit with instructions on how to take a follow-up sample and a pre-paid return envelope addressed to the NRL for Enteropathogenic bacteria at NIPH. The follow up sample was requested to be taken approximately five weeks after the date of the initial infection detected (date of initial sample received at NRL confirming the infection). In case of non-response, one reminder was sent by post. NIPH developed the questionnaire and was responsible for carrying out the study. The survey questionnaire used is provided in Online Resource 1.

Through the questionnaire, participants provided information about demographics, past medical history, regular medication use, medical treatment of the acute *Salmonella* infection, diet and pre- or probiotic consumption. The questionnaire also included detailed questions about exposures from one week prior to the detection of infection to the time of provision of the follow-up sample (five weeks after the initial infection was detected). Details on exposures assessed are provided in the statistical analysis section below. Information regarding symptoms and hospitalisation was also collected.

### Laboratory analyses and data

The received samples were cultured following standard *Salmonella* isolation protocol and bacterial identification was confirmed using Matrix-Assisted Laser Desorption Ionization Time of Flight Mass Spectrometry (MALDI-TOF MS). If a positive *Salmonella* was identified, the isolates was prepared for Whole Genome Sequencing (Illumina) using a standard in-house WGS protocol, in short:

DNA was extracted using MagNAPure 96 (Roche Molecular Systems Inc., Pleasanton, US), and library preparation was performed with KAPA HyperPlus (Kapa Biosystems, Wilmington, US). Adapter dimers were removed by Agencourt AMPure XP (Beckmann Coulter Life Sciences, Indianapolis, US), and Illumina technology (MiSeq or NextSeq, Illumina, Inc., San Diego, US) was used to perform paired-end (250 bp x2) sequencing aiming for coverage of > 50x. FastQC (Babraham Bioinformatics, United Kingdom, Cambridge) were used for quality control of the raw reads. Kraken was used for species identification. All sequences have been submitted to the European Nucleotide Archive (ENA) and are available through BioProject PRJEB76788.

Sequence assemblies were performed within Ridom SeqSphere + software, using the Velvet assembly algorithm. First, multi-locus sequence typing (MLST) and core genome multi-locus sequence typing (cgMLST) using the EnteroBase schema for *Salmonella* enterica were performed, followed by phylogenetic analysis by minimum spanning tree (pairwise ignore missing values) and screening for antimicrobial resistance determinants (NCBI AMRFinderPlus). Sequences with less than 90% good targets in the cgMLST schema were excluded from further analysis. Finally, SeqSero was used to classify Serotypes.

Each initial isolate received at the laboratory was given a unique identifier enabling linkages with the survey data. The laboratory data were linked with the survey data before the dataset was anonymised. Laboratory data collection was performed using Microsoft Excel and Ridom SeqSphere+.

### Definition of long-term or short-term shedding infections

We classified participants as long-term (LTS) or short-term (STS) shedders/shedding infections of NTS based on the initial and 5-week follow-up sample results. We classified a participant as having LTS phenotype if the initial sample was identical to the follow-up sample (≤ 5 allelic differences, based on cgMLST), and having STS phenotype if no *Salmonella* spp. was isolated in the follow-up sample. If a different *Salmonella* strain was detected in the follow-up sample (>5 allelic differences based on cgMLST) the infection was classified as a new infection, and the participant was excluded from further analyses. Additionally, genotypic resistance of an isolate to a class of antibiotic was defined as the presence of a genetic determinant known to confer antimicrobial resistance to that class of antibiotic.

### Statistical analysis

#### Description of participants

We described study participants in terms of demographic characteristics and the microbiological characteristics of their isolated *Salmonella* spp. and presented their distribution by LTS and STS. We assessed the representativeness of study participants (included cases) by comparing their demographic characteristics with those of all salmonellosis cases notified to MSIS during the study period (see Online Resource 2, Table S1).

#### Assessing ris for long-term shedding infectionsk factor

We presented the overall distribution of participants according to LTS and STS categorisation in terms of demographics, *Salmonella* serotype, antimicrobial resistance, and other factors considered as potential risk factors for LTS. We calculated adjusted odds ratios (aORs) with 95% confidence intervals (95%CIs) using logistic regression (i.e., case-case study) to examine the association between potential risk factors associated with long-term shedding. 

Potential risk factors included among others: age-group, working place (for participants 16 years or older, e.g. work at health care institutions, day-care, café/restaurants), household size, member in household below 6 years old, *Salmonella* serotype, having a chronic disease, regular use of medication, diet, pre- or probiotic consumption (sour milk or yogurt with probiotics, or pre- or probiotics in drop, tablet or powder form), intake of regular medication, medication received specifically as treatment for salmonellosis, history of abdominal surgery (at any point in life before infection), travel abroad and contact with animals. Several of these factors were assessed based on the hypothesis that an individual’s living or working environment could influence long-term shedding. This influence could occur through mechanisms such as continued exposure to contaminated environments, reinfection with the same strain, or ongoing transmission between individuals in close contact settings. For example, one main exposure we assessed was contact with animals both during the week before detection of infection and after recovering from the infection, as animals are known to potentially shed high loads of *Salmonella* into their environment. This environmental contamination can persist even after the individual has recovered, potentially leading to reinfection which would inaccurately lead to categorisation of this infection as LTS.

Model selection for the multivariable regression was conducted using the likelihood ratio test and the Akaike Information Criterion. We checked for interactions between our co-variates by including interaction terms in our models. We considered a p-value of ≤ 0.05 as statistically significant.

#### Symptoms, hospitalisation, and antimicrobial resistance

We described symptoms during infection and at the time of follow-up sampling (five weeks after initial detection) and hospitalisations reported by STS and LTS participants. We also compared the distribution of participants with STS and LTS by antibiotic/genotypic resistant. Chi-square test was used to assess differences in the distributions with different characteristics and Mann-Whitney (rank sum) test was used to assess any potential difference in the median time of hospitalisation among STS vs. LTS.

Statistical analysis was performed on anonymised data using STATA version 16 (Stata Corporation, College Station, TX, USA).

## Results

### Description of participants

**Fig. 1:**
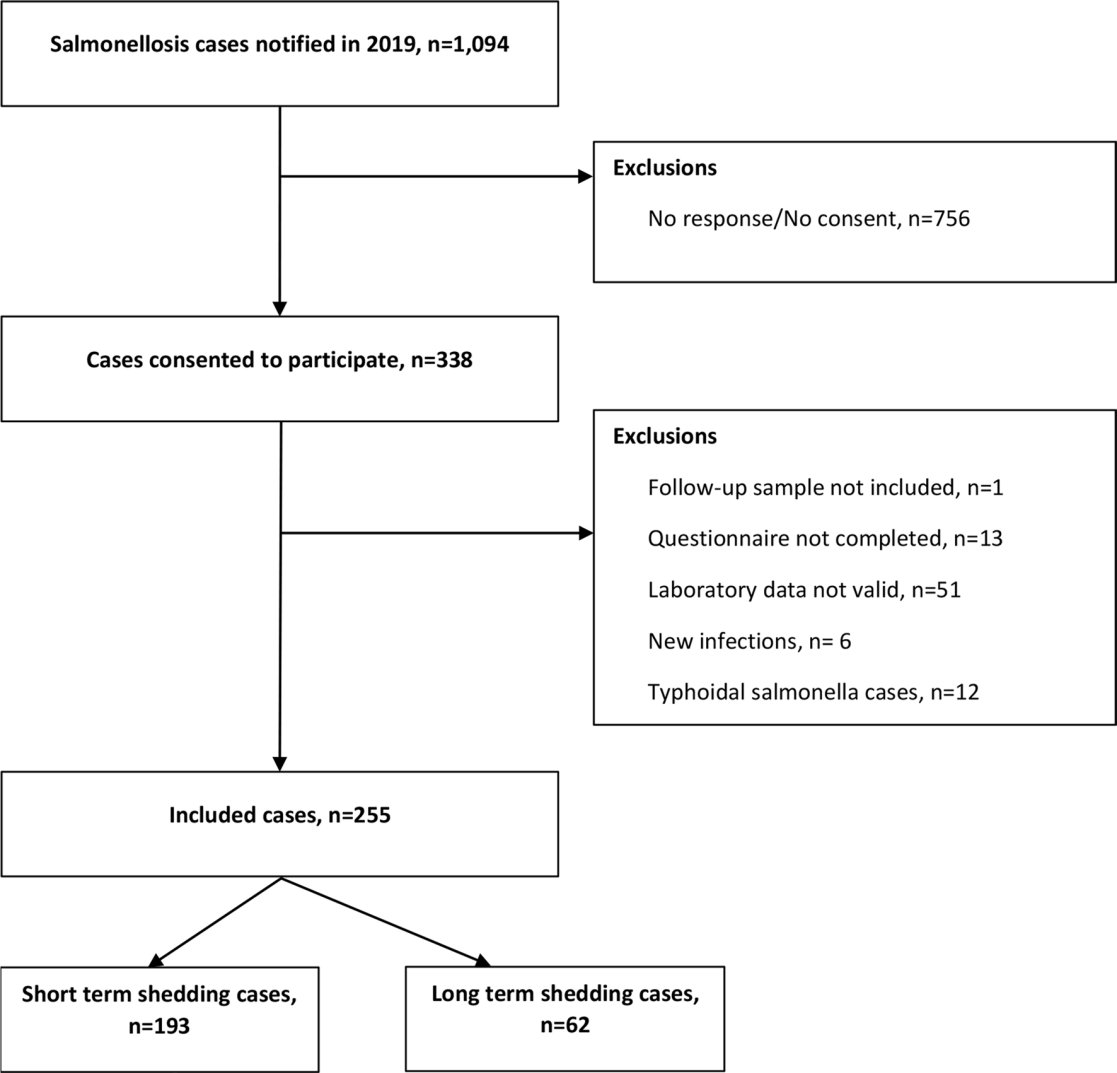
Inclusion summary flowchart, MoMIR study, Norway, 2019

During the study period, a total of 1,094 cases of salmonellosis were notified to MSIS, and all were invited to participate in the study. Of these, 338 (31%) gave their consent to participate, of which 25% (*n* = 83) were excluded because: (i) they did not send a follow-up stool sample, (ii) did not complete the questionnaire, (iii) the laboratory data was not valid, (iv) the follow-up sample indicated a new infection, or (v) the laboratory result was a typhoidal *Salmonella*. Further details are presented in the inclusion summary flowchart in Fig. 1.

After applying the exclusion criteria, 255 individuals were included in the study, resulting in 23% participation rate. After analysis of the genomes of the initial isolates and the follow-up samples, 62 participants (24%) were classified as LTS, and 193 (76%) as STS. Age was reported by 250 (98%) of the participants, ranging from 1 to 91 years old; median age 51 years (IQR: 25). Participants from all counties of Norway replied to the survey, with 13% of them having residency in Trøndelag, 13% in Oslo, and 11% Vestland. When comparing the proportions of the participant demographic characteristics with those of all *Salmonella* cases notified in 2019, study participants were representative by age, sex and county of residence with a slightly overrepresentation of older adults, 40–69 years old (see Online Resource 2, Table S1).

### Serotypes identified

*S*. *Enteritidis* was the most common serotype identified in 42% (*n* = 107) of the isolates, followed by *S*. *Typhimurium* in 11% (*n* = 27) and *S. Agbeni or Bron* in 9% (*n* = 22). During the study period, an outbreak of *Salmonella Agbeni* or *Bron* (22 cases) linked to imported dried fruits occurred in Norway. Among these serotypes, *S*. *Enteritidis* and *S. Typhimurium* showed a 21% (23/107) and 19% (5/27) LTS frequency, respectively. The highest percentage of LTS was 45% (10/22) among participants identified with *S. Agbeni or Bron*, followed by 29% (2/7) among *S. Coeln* and 25% (4/16) among monophasic *S. Typhimurium* cases. The lowest percentage of LTS was 11% (1/9) among *S. Newport* cases.

### Assessing risk factors for long-term shedding infection

Some of the main risk factors that were assessed for the risk of LTS before reaching the final multivariable model are included in Tables 1 and 2 (further details on subcategories and additional factors provided in Online Resource 2, Table S2 and S3. Due to the length of the questionnaire and the large number of factors assessed, only a subset of the main risk factors is presented in the main article. A complete list of all questions included, and factors assessed can be found in the study questionnaire (Online Resource 1).

**Table 1:** Partial results from univariable analysis assessing risk factors for long-term shedding infections of non-typhoidal *Salmonella*. Here some of the risk factors that were assessed before reaching the final multivariable model are presented

		Total cases (*n* = 255)		Short-term shedding cases (*n* = 193)		Long-term shedding cases (*n* = 62)		Univariable analysis	
								Odds Ratio	95% Confidence Interval
		*n*	%	*n*	%	*n*	%		
**Household size**	1 person (live alone)	34	13%	28	82%	6	18%	Ref	Ref
	2 persons	110	43%	81	74%	29	26%	1.67	0.63–4.44
	3–5 persons	106	42%	80	75%	26	25%	1.52	0.57–4.07
	> 5 persons	5	2%	4	80%	1	20%	1.17	0.11–12.4
**Children < 6 years in household**	No	186	73%	140	75%	46	25%	Ref	Ref
	Yes	32	13%	23	72%	9	28%	1.19	0.51–2.76
	Missing^*#*^	37	14%	30	81%	7	19%		
**Working in kindergarten or nursery school (only for participants > 16 years old)**	No	213	93%	164	77%	49	23%	Ref	Ref
	Yes	7	3%	5	71%	2	29%	1.34	0.25–7.12
	Missing^*#*^	9	4%	7	78%	2	22%		
**Working in hospital**,** nursing home or other health institution (only for participants > 16 years old)**	No	187	82%	143	76%	44	24%	Ref	Ref
	Yes	35	15%	28	80%	7	20%	0.81	0.33–1.99
	Missing^*#*^	7	3%	5	71%	2	29%		
**Working in café**,** restaurant**,** or other serving place (only for participants > 16 years old)**	No	213	93%	166	78%	47	22%	Ref	Ref
	Yes	8	3.5%	4	50%	4	50%	3.53	0.85–14.7
	Missing^*#*^	8	3.5%	6	75%	2	25%		
**Animal contact during the week before having** ***Salmonella*** **infection detected**	No	158	62%	120	76%	38	24%	Ref	Ref
	Yes	82	32%	62	76%	20	24%	1.09	0.55–1.90
	Missing/don’t know^*#*^	15	6%	11	73%	4	27%		
**Animal contact after recovering from** ***Salmonella*** **infection**	No	107	42%	79	74%	28	26%	Ref	Ref
	Yes	135	53%	105	78%	30	22%	0.81	0.45–1.46
	Missing^*#*^	13	5%	9	69%	4	31%		
**Work-related animal contact**	No	245	96%	185	76%	60	24%	Ref	Ref
	Yes	7	3%	6	86%	1	14%	0.51	0.0-4.35
	Missing^*#*^	3	1%	2	67%	1	33%		
**Travel abroad during the week before having** ***Salmonella*** **infection detected**	No	96	38%	68	70%	28	29%	Ref	Ref
	Yes	156	61%	123	79%	33	21%	0.65	0.36–1.17
	Missing^*#*^	3	1%	2	67%	1	33%		
**Use of regular medication** **(Not salmonellosis related)**	No	115	45%	95	83%	20	17%	Ref	Ref
	Yes	138	54%	97	70%	41	30%	2.01*	1.10–3.68
	Missing^*#*^	2	0.8%	1	50%	1	50%		
**Any medical treatment of acute** ***Salmonella*** **infection received (selection from the list below)**	No	40	16%	35	88%	5	12%	Ref	Ref
	Yes	215	84%	158	73%	57	27%	2.53	0.94–6.76
Natural remedies	No	255	100%	193	76%	62	24%	Ref	Ref
	Yes	0	0%	0	0%	0	0%	- ^*†*^	- ^*†*^
Antibiotics	No	190	75%	148	78%	42	22%	Ref	Ref
	Yes	65	25%	45	69%	20	31%	1.57	0.84–2.94
Diarrheal medication	No	166	65%	127	77%	39	23%	Ref	Ref
	Yes	89	35%	66	74%	23	26%	1.13	0.63–2.06
Food supplements	No	228	89%	177	78%	51	22%	Ref	Ref
	Yes	27	11%	16	59%	11	41%	2.39*	1.04–5.46
Other medication	No	242	95%	181	75%	61	25%	Ref	Ref
	Yes	13	5%	12	92%	1	8%	0.25	0.03–1.94
Pain medication	No	84	32%	65	77%	19	23%	Ref	Ref
	Yes	171	67%	128	75%	43	25%	1.15	0.62–2.13
Use of pre- or probiotics	No	176	69%	136	77%	40	23%	Ref	Ref
	Yes	79	31%	57	72%	22	28%	1.31	0.72–2.40
Alternative treatment (e.g. homeopathy, acupuncture)	No	251	98%	191	76%	60	24%	Ref	Ref
	Yes	4	2%	2	50%	2	50%	3.18	0.44–23.1
**No medication for** ***Salmonella*** **infection**	No	200	78%	148	74%	52	26%	Ref	Ref
	Yes	55	22%	45	82%	10	18%	0.63	0.30–1.35
**History of abdominal surgery prior to infection**	No	209	82%	161	77%	48	23%	Ref	Ref
	Yes	45	18%	31	69%	14	31%	1.51	0.75–3.08
	Missing^*#*^	1	0.4%	1	100%	0	0%		

*:P-value < 0.05

*#: Missing values were not included in univariable and multivariable analysis*

*†: Estimates could not be provided as no participants were exposed to the assessed potential risk factor*

**Table 2:** Results from multivariable analysis, assessing risk factors for long-term shedding infections of non-typhoidal *Salmonella*

	Total cases, *n* = 255	Short-term shedding cases, *n* = 193	Long-term shedding cases, *n* = 62	Univariable analysis		Multivariable analysis	
	*n* (%)	*n* (%)	*n* (%)	Odds Ratio	95% Confidence Interval	Adjusted Odds Ratio	95% Confidence Interval
**Age group (years)**							
0–5	15 (6%)	8 (4%)	7 (11%)	4.11*	(1.21–13.97)	6.71*	(1.67–26.94)
6–17	15 (6%)	12 (6%)	3 (5%)	1.18	(0.28–4.95)	0.84	(0.14–5.10)
18–44	57 (22%)	47 (24%)	10 (16%)	Ref	Ref	Ref	Ref
45–64	119 (47%)	91 (47%)	28 (45%)	1.45	(0.65–3.23)	1.69	(0.70–4.07)
>=65	44 (17%)	33 (17%)	11 (18%)	1.57	(0.60–4.11)	1.27	(0.44–3.69)
Missing^a^	5 (2%)	2 (1%)	3 (5%)				
**Regular Medication use** ^**b**^							
No	115 (45%)	95 (49%)	20 (32%)	Ref	Ref	Ref	Ref
Yes	138 (54%)	97 (50%)	41 (66%)	2.01*	(1.10–3.68)	2.17*	(1.02–4.64)
Missing^a^	2 (1%)	1 (1%)	1 (12%)				
**Lactose free diet**							
No	247 (96%)	190 (98%)	57 (92%)	Ref	Ref	Ref	Ref
Yes	8 (3%)	3 (2%)	5 (8%)	5.56*	(1.29–23.96)	7.24*	(1.48–35.40)
**Diet with use of “other pre- or probiotic products”** ^**c**^							
No	248 (97%)	190 (98%)	58 (94%)	Ref	Ref	Ref	Ref
Yes	7 (3%)	3 (2%)	4 (6%)	4.37	(0.95–20.08)	8.68*	(1.39–54.30)
**Serotype group**							
*Enteritidis*	107 (42%)	84 (44%)	23 (37%)	1.21	(0.41–3.53)	1.82	(0.50–6.57)
*Typhimurium*	27 (11%)	22 (11%)	5 (8%)	Ref	Ref	Ref	Ref
*Agbeni or Bron*	22 (9%)	12 (6%)	10 (16%)	3.67*	(1.02–13.23)	6.29*	(1.40-28.16)
*Monophasic Typhimurium*	16 (6%)	12 (6%)	4 (6%)	1.47	(0.33–6.51)	2.54	(0.44–14.50)
*Stanley*	14 (5%)	11 (6%)	3 (5%)	1.20	(0.24–5.97)	2.16	(0.36–13.04)
*Newport*	9 (4%)	8 (4%)	1 (2%)	0.55	(0.06–5.46)	1.39	(0.12–15.84)
*Coeln*	7 (3%)	5 (3%)	2 (3%)	1.76	(0.26–11.84)	3.34	(0.40-28.17)
Other	53 (21%)	39 (20%)	14 (23%)	1.58	(0.50–4.97)	2.73	(0.68–10.96)

*: P-value < 0.05

*a: Missing values were not included in univariable and multivariable analysis*

*b: Regular medication: not related (not as part of treatment) to the acute gastrointestinal infection*

*c: Use “other pre or probiotic products” in their general diet (sour milk or yogurt with probiotics*,* or pre- or probiotics in drop*,* tablet or powder form)*,* not as part of the treatment for the acute gastrointestinal infection (this variable is different than the one presented in* Table 1*)*

Among the 255 participants, around 32% (*n* = 82) reported animal contact up to a week before having the infection detected and 53% (*n* = 135) after recovering from the disease. More than half (61%, *n* = 156) of the participants reported travel abroad the last week before having the infection detected (Table 1).

About half (54%, *n* = 138) of the participants received regular medication (not related to *Salmonella*), including 13% (*n* = 33) using antacids, 9% (*n* = 24) food supplements, 2% (*n* = 4) using corticosteroids, and 45% (*n* = 116) “other regular medication” (Online Resource 2, Table S3). 18% (*n* = 45) had a history of abdominal surgery prior to the infection, and 84% (*n* = 215) reported having received medication in the acute phase of the *Salmonella* infection. Medication received during the acute infection included among others, diarrheal medication (*n* = 89, 35%), use pre-or probiotics (*n* = 79, 31%) and antibiotics (*n* = 65, 25%) (Table 1).

Moreover, 3% (*n* = 8) of participants reported having a lactose free diet (not related to infection), and none of them reported being vegetarian, vegan or pescetarian (Online Resource 2, Table S2). Also, 33% participants (*n* = 76) used pre- or probiotics in their diet. When clarifying what type of pre or probiotics they used, 27% (*n* = 68) indicated sour milk or yogurt with probiotics, 5% (14) drops, tablets or powder with pre-or probiotics, and 3% (*n* = 7) used “other pre- or probiotic products”.

In our multivariable analysis (Table 2), we observed that participants in age group 0–5 years were 6.7 times more likely to shed *Salmonella* five weeks after infection (LTS) compared to participants in age group 18–44 years (aOR = 6.71, 95%CI: 1.67–26.94). Participants using regular medication, not related to the acute gastrointestinal infection, were two times more likely to be LTS (aOR = 2.17, 95%CI: 1.02–4.64) compared to those who did not. However, when we explored the detailed questions about regular medication in the univariable analysis, “corticosteroids” and “other medication” was associated with increased odds, but in our multivariable analyses (excluding the overall “regular medication” variable in this analysis), none of the single available options in the questionnaire (antacids, corticosteroids, immunosuppressives, insulin, food supplements etc.) were associated with increased risk of LTS. Also, participants who reported having a lactose free diet or used “other pre- or probiotic products” in their diet (even though the numbers are small) were more likely to be categorised as LTS compared to those who did not, with aOR 7.24 (95%CI: 1.48–35.40) and 8.68 (95%CI: 1.39–54.30) respectively. Regarding serotypes, the participants identified with *S. Agbeni or Bron* were 6 times more likely to be categorised as LTS compared to the participants with *S. Typhimurium* (aOR = 6.29, 95%CI:1.40–28.16).

We should note that the use of “other pre- or probiotic products” in their diet, reported by 3% of participants, was not associated with higher odds of LTS in the univariable analysis (OR:4.37, 95%CI: 0.95 – 20.08) but became significant when included in the multivariable analysis (aOR: 8.68, 95%CI: 1.39–54.30). Among the rest of the assessed risk factors, receiving food supplements as part of the medical treatment for the NTS infection, reported by 11% of participants, was associated with higher risk of LTS in the univariable (OR:2.39, 95%CI: 1.04–5.46), but this association was not significant in the multivariable analysis.

### Symptoms

#### Symptoms during infection

Only four participants (1.6%) were asymptomatic (3 STS and 1 LTS). The most frequent symptoms reported by the participants during their infection were diarrhea (90%), abdominal pain (67%) and fever (64%). Other common symptoms were nausea (39%), mucus in stool (36%), blood in stool (24%) and vomiting (23%) (Table 3). No difference was observed in the distribution of symptoms among participants with STS and LTS (chi square p-values > 0.05).

**Table 3:** Symptoms during infection as reported by study participants and evaluated based on shedding phenotype of non-typhoidal *Salmonella*

Reported symptom	Total cases (*n* = 255)		Short-term shedding cases (*n* = 193)		Long-term shedding cases (*n* = 62)	
	*n*	%	*n*	%	*n*	%
Diarrhea	230	90%	174	90%	56	90%
Abdominal pain	172	67%	133	69%	39	63%
Fever	162	64%	119	62%	43	69%
Nausea	99	39%	73	37%	26	41%
Mucus in stool	92	36%	66	34%	26	42%
Blood in stool	61	24%	46	24%	15	24%
Vomiting	59	23%	43	22%	16	26%
Joint pain	54	21%	40	21%	14	23%
Other symptoms	51	20%	41	21%	10	16%

#### Prolonged symptoms

At the time of the second stool sample collection, approximately five weeks after the initial sample, 35 participants (14%) reported experiencing symptoms. The most common prolonged symptoms were abdominal pain (*n* = 15, 6%), mucus in stool (*n* = 12, 5%), and diarrhea (*n* = 9, 4%) (see Online Resource 2, Table S4 for further details). Duration for the reported symptoms could not be determined based on our questionnaire. No difference was observed in the distribution of prolonged symptoms between participants with STS and LTS (chi square p-values > 0.05).

### Hospitalisation

31% of the participants (*n* = 80) were hospitalised, 30% among those with STS and 37% among those with LTS (no difference observed, chi square p-value = 0.264). Among 79 of the hospitalised participants with available information, reported duration of hospital stay ranged from 1 to 18 days, median = 4 days (IQR:3). No difference was observed in the median duration of hospitalisation among LTS and STS (rank-sum test p-value = 0.122). Moreover, no difference was observed in the distribution of hospitalisations among participants infected with different *Salmonella* serotypes (chi square p-value = 0.824). Further details regarding hospitalisations by serotype and by short-term shedder and long-term shedder phenotypes are presented in Online Resource 2, Table S5.

### Antimicrobial resistance

The in-silico analysis of AMR determinants from the *Salmonella* isolates recovered from the participants, identified Quinolone resistance as the most frequent AMR genotype with 16% (*n* = 41), followed by tetracycline (*n* = 35, 14%) and resistance to β-lactams (*n* = 33, 13%). Only one isolate was identified with a Lincosamide resistance genotype. A multi-drug resistance genotype (resistance to more than three antibiotic classes) was identified in 30 isolates (12%), however only a single isolate was predicted to have an ASSuT phenotype. Of all the investigated antimicrobial families and genotypes, only Fosfomycin resistance was significantly associated with LTS phenotype (chi-square p-value = 0.017, n=4) (Table 4).

**Table 4:** Distribution of detected antibiotic resistant genotypes in participants by short-term shedder and long-term shedder phenotypes of non-typhoidal *Salmonella*

	Resistant isolates						Chi square test *p*-value
	Short-term shedders (*n* = 193)		Long-term shedders (*n* = 62)		Total (*n* = 255)		
**Antibiotic, genotypic resistant**	***n***	%	***n***	%	***n***	%	
Aminoglycoside	22	11%	7	11%	29	11%	0.981
β-Lactam	25	13%	8	13%	33	13%	0.992
Fosfomycin	1	0.5%	3	4.8%	4	1.6%	0.017
Lincosamide	1	0.5%	0	0%	1	0.4%	0.570
Chloramphenicol	5	2.6%	0	0%	5	2%	0.201
Florfenicol	3	1.6%	0	0%	3	1.2%	0.323
Quinolone	31	16%	10	16%	41	16%	0.990
Streptogramin	0	0%	0	0%	0	0%	-
Sulfonamide	22	11%	6	9.7%	28	11%	0.706
Tetracycline	27	14%	8	13%	35	14%	0.829
Trimethoprim	6	3%	0	0%	6	2.4%	0.160
MDR* (≥ 3)	24	12%	6	9.7%	30	12%	0.558
ASSuT**	1	0.5%	0	0%	1	0.4%	0.507

*Multi drug resistance, resistance to more than 3 classes of Antimbiotics

** Resistant to Ampicillin (A), Streptomycin(S), Sulphonamide (Su), Tetracycline (T)

## Discussion

In our study, we investigated various potential risk factors associated with LTS among NTS infections. We found that young age, regular medication use, lactose-free diet, and specific *Salmonella* serotypes were associated with increased odds of LTS. Understanding the dynamics of *Salmonella* shedding and clearance continues to be a complex area of study, with numerous interrelated and contributing factors [10, 17]. Studies exploring potential risk factors associated with LTS among NTS infections in humans are sparse.

In our study we observed that 24% of the participants in our study continued to shed *Salmonella* even five weeks after detection of the infection, which is significantly higher than what has previously been reported. Previous observations reported that non-typhoidal *Salmonella* serovars, such as *S. Typhimurium*, typically cause self-limiting infections where no shedding can be detected within 12 to 28 days [15]. Longer shedding is rare with only 2.2% of cases shedding NTS beyond 30 days [13] and fewer than 1% become carriers beyond a year after infection [5, 12]. This high proportion of LTS observed in Norway raise potential concerns about prolonged transmission risks from infected individuals, and the need for effective infection control measures.

Age was found to play a significant role in persistence. Children under five years old were nearly seven times more likely to be LTS carriers (aOR: 6.70, 95% CI: 1.67–26.94) compared to adults aged 18–44, while no significant differences were observed for those over 45 years old. This is in line with previous studies that have shown that younger age groups shed bacteria for longer periods, while older age groups clear the infection faster [12].

We also found that *Salmonella* serotype *Agbeni* or *Bron* was associated with higher odds of LTS compared with the Typhimurium serotype. No specific virulence genes were found to be significantly associated with LTS. Several serotypes of the *Salmonella* strain have previously been associated with increased odds of LTS however the *Agbeni* or *Bron* serotype, which is rare, has not been indicated [13]. We should note that an outbreak of *Salmonella Agbeni* or *Bron* (22 cases) linked to imported dried fruits occurred during the study period in Norway [18], and samples from this outbreak were included in the study. We decided to not exclude these cases from our study, since the cases represent a small proportion of the study cases (9%), and the variability in case characteristics made it valuable to explore the potential association between this rare serotype and LTS. However, further investigation is needed, as our findings may reflect the specific impact of the outbreak strain rather than the serotype itself. Additionally, we performed a sensitivity analysis where we excluded the outbreak cases (all Agbeni or Bron cases) and our results were robust for the remaining risk factors associated with LTS, and the proportion of LTS phenotype among included NTS cases was 22%.

Furthermore, we found that a lactose-free diet was associated with LTS, suggesting that dietary factors may influence the persistence and duration of shedding. This is in line with previous research showing that a two-weeks dietary intervention with lactic acid fermented products significantly reduced enteric pathogens in stool samples of children [19]. At the same time, 33% of the study participants reported using pre- or probiotics in their diet, but no association was found with LTS. However, when examining specific subcategories of pre- or probiotic use, the consumption of “other pre- or probiotic products” was found to be associated with LTS. The strength of this finding is limited by the small number of participants in this subgroup (*n* = 7) and the difficulty in identifying which specific “other pre- or probiotic products” were used. Some studies suggest that probiotics may help reduce *Salmonella* colonisation and shedding in various animal species [20–22]. However, it is important to note that direct evidence linking pre- or probiotic use to reduced long-term shedding in human *Salmonella* infections is limited. Further research is needed to clarify their potential role in humans.

Antibiotic use has been linked to extended excretion periods of many enteric pathogens including *Salmonella.* In our study we observed 69% and 78% clearance rates within five weeks among those who received antibiotics during their infection and those who did not respectively. However, no significant difference was found between antibiotic use and LTS, which indicates that the potential prolonged shedding did not exceed five weeks to an extent that was measurable in this study. Overall, the strains analysed in this study had a very low level of antimicrobial resistance, and only resistance to Fosfomycin was found to be associated with LTS, but the small number of cases with Fosfomycin resistance (n = 4) limits the importance of this finding.

Moreover, we found that participants who were receiving regular medication (unrelated to *Salmonella* infection) were twice as likely to exhibit LTS. When we looked at the subcategories included within “regular medication use”, almost half of the participants (45%) reported receiving “other medications” without any further specification (see Online Resource 2, Table S3) making this finding hard to evaluate. Taking other regular medication could be an indication of comorbidities or general poorer health which in turn could contribute to LTS, but this warrants further investigation.

Interestingly, our analysis did not identify a significant association between abdominal surgery prior to infection—including gallbladder surgery—or its subcategories (see Online Source 2, Table S3) and LTS. Although, previous studies have reported that gallstones can harbor *Salmonella* biofilms [23], and gallbladder removal has been performed/used as treatment of chronic carriers [24].

A limitation we could not rule out in our study is the potential misclassification of cases as LTS when they may have been reinfections with the same strain. This could have led to an overesti-mation of the proportion of LTS. We evaluated participants’ living and working environments as potential risk factors for long-term shedding, hypothesizing that continued exposure to contaminated environments, or ongoing transmission in close-contact settings that could result in reinfection with the same strain might contribute to prolonged shedding. However, we did not find a significant association between environmental factors (e.g., animal contact, workplace conditions) and LTS. This lack of association suggests that reinfection with the same strain was unlikely among our study participants.

In conclusion, we found a much higher proportion of LTS phenotype among included NTS cases than reported in the current literature. Also, some factors associated with LTS were identified, but further research is needed to increase knowledge about the mechanisms behind LTS and related risk factors that could potentially lead to informing infection control and preventive measures against *Salmonella* infections in Norway and globally.

## Electronic supplementary material

Below is the link to the electronic supplementary material.

Supplementary Material 1

Supplementary Material 2

## Data Availability

No datasets were generated or analysed during the current study.
