# Supplementary material for: Risk factors associated with long-term shedding infections of non-typhoidal Salmonella in humans
Source: Eur J Clin Microbiol Infect Dis. 2025 May 27;44(9):2047–57. doi: 10.1007/s10096-025-05165-x (PMC12457537; doi:10.1007/s10096-025-05165-x)
Supplement: Supplementary file 1 — Supplementary Material 1 [file 10096_2025_5165_MOESM1_ESM.pdf]

Spørreskjema [v2]

Side 1

Spørreskjema til studien: «Hvorfor skiller noen ut salmonellabakterier lenge etter at de har blitt friske?»

Hjertelig takk for at du har valgt å bli med i studien og tar tiden med å fylle ut spørreskjemaet!

Alle spørsmål i spørreskjemaet gjelder for den som er syk. Dersom du fyller ut på vegne av noen under 16 år, så ønsker vi svar som gjelder barnet og ikke for den som hjelper til med å fylle ut skjemaet. Dersom du selv er yngre enn 16 år, er det fint om foreldre eller andre foresatte hjelper deg med å fylle ut skjemaet.

Vennligst fyll ut deltakernummer (finnes på invitasjonsbrevet): *

Har deltakeren fylt 16 år? *

- Ja
- Nei

Deltakerens fødselsår (4 siffer): *

Innledende spørsmål

1. Hvor mange personer er det i husholdningen der du bor, deg selv medregnet? *

- 1 person (bor alene)
- 2 personer
- 3-5 personer
- 6 eller flere

Dette elementet vises dersom et av følgende alternativer er valgt på spørsmål «1. Hvor mange personer er det i husholdningen der du bor, deg selv medregnet?»: 2 personer, 3-5 personer, 6 eller flere

Er noen av disse personene barn under 6 år? *

- Ja
- Nei

Dette elementet vises dersom et av følgende alternativer er valgt på spørsmål «Har deltakeren fylt 16 år?»: Ja

Hva er din høyeste fullførte utdannelse? *

- Jeg har ikke fullført grunnskole
- Grunnskole, ungdomsskole, realskole
- Videregående, gymnas, fagutdannelse og lignende
- Universitet eller høgskole
- Annen utdannelse

Dette elementet vises dersom et av følgende alternativer er valgt på spørsmål «Hva er din høyeste fullførte utdannelse?»: Annen utdannelse

Vennligst spesifiser utdanningen: *

Dette elementet vises dersom et av følgende alternativer er valgt på spørsmål «Har deltakeren fylt 16 år?»: Ja

Går du eller arbeider du i barnehage, barnepark eller daghjem? *

- Ja
- Nei

Dette elementet vises dersom et av følgende alternativer er valgt på spørsmål «Har deltakeren fylt 16 år?»: Ja

Bor eller jobber du på sykehjem, sykehus eller annen helseinstitusjon? *

- Ja
- Nei

Dette elementet vises dersom et av følgende alternativer er valgt på spørsmål «Har deltakeren fylt 16 år?»: Ja

Jobber du på kafé, restaurant eller annet serveringsted? *

- Ja
- Nei

Side 2

Sideskift

2. Spørsmål om salmonellainfeksjonen

Du har fått påvist infeksjon med salmonellabakterien og her følger noen spørsmål om sykdommen

Når startet sykdommen? Dato du første gang merket symptomene *

Hvis du er usikker, oppgi omtrentlig dato.

dd.mm.åååå

Hvilke symptomer hadde du? *

Du kan krysse av for flere alternativer:

- Kvalme
- Magesmerter
- Blod i avføringen
- Oppkast
- Leddsmerter
- Slim i avføringen
- Diaré (3 eller flere løse avføringer per døgn)
- Feber (38 grader eller mer)
- Andre symptomer
- Ingen symptomer

Dette elementet vises dersom et av følgende alternativer er valgt på spørsmål «Hvilke symptomer hadde du?»: Andre symptomer

Du har krysset av 'Andre symptomer'. Kan du beskrive hvilke? *

Er du frisk fra salmonellainfeksjonen nå? *

- Ja
- Nei
- Usikker

Dette elementet vises dersom et av følgende alternativer er valgt på spørsmål «Er du frisk fra salmonellainfeksjonen nå?»: Nei, Usikker

Hvilke symptomer har du fortsatt? *

Du kan krysse av for flere alternativer:

- Kvalme
- Magesmerter
- Blod i avføringen
- Oppkast
- Leddsmerter
- Slim i avføringen
- Diaré (3 eller flere løse avføringer per døgn)
- Feber (38 grader eller mer)
- Andre symptomer
- Ingen symptomer

Dette elementet vises dersom et av følgende alternativer er valgt på spørsmål «Hvilke symptomer har du fortsatt?»: Andre symptomer

Du har krysset av 'Andre symptomer'. Kan du beskrive hvilke? *

Dette elementet vises dersom et av følgende alternativer er valgt på spørsmål «Er du frisk fra salmonellainfeksjonen nå?»: Nei, Usikker

Har du nylig blitt påvist salmonellabakterier i avføringen igjen? *

- Ja
- Nei

Dette elementet vises dersom et av følgende alternativer er valgt på spørsmål «Har du nylig blitt påvist salmonellabakterier i avføringen igjen?»: Ja

Når ble salmonellabakteriene påvist igjen? *

dd.mm.åååå

Dette elementet vises dersom et av følgende alternativer er valgt på spørsmål «Er du frisk fra salmonellainfeksjonen nå?»: Ja

Hvor mange dager var du syk? *

Hvis du er usikker, oppgi omtrentlig antall dager.

Ble du innlagt på sykehus for denne sykdommen? *

- Ja
- Nei

Dette elementet vises dersom et av følgende alternativer er valgt på spørsmål «Ble du innlagt på sykehus for denne sykdommen?»: Ja

Hvor mange dager var du innlagt? *

Hvis du er usikker, oppgi omtrentlig antall dager.

Hadde andre personer i husholdningen der du bor diarésykdom i perioden fra du ble syk fram til nå? *

- Ja
- Nei
- Vet ikke

Dette elementet vises dersom et av følgende alternativer er valgt på spørsmål «Hadde andre personer i husholdningen der du bor diarésykdom i perioden fra du ble syk fram til nå?»: Ja

Fikk vedkommende påvist salmonellainfeksjon? *

- Ja
- Nei

Tok du noen av følgende medikamenter mens du hadde salmonellainfeksjonen? *

Du kan krysse av for flere alternativer:

- Smertestillende/febernedsettende medisiner (paracet, ibux, dispril og lignende)
- Medisiner mot diaré (imodium, travello og lignende)
- Antibiotika som penicillin og lignende
- Naturmedisin
- Helsekost, kosttilskudd og vitaminer
- Pre- eller probiotika (f.eks. syrnet melk eller yoghurt som Activia, Actimelk, BioQ, Biola, Cultura eller i dråpe-, tablett- eller pulverform)
- Alternativ behandling (f.eks. homeopati, akupunktur)
- Andre medisiner mot salmonellainfeksjonen
- Nei, jeg tok ingen medisiner mot salmonellainfeksjonen

Dette elementet vises dersom et av følgende alternativer er valgt på spørsmål «Tok du noen av følgende medikamenter mens du hadde salmonellainfeksjonen?»: Andre medisiner mot salmonellainfeksjonen, Naturmedisin, Alternativ behandling (f.eks. homeopati, akupunktur)

Hvilke medikamenter eller behandlinger tok du? *

Sideskift

Side 3

3. Helsetilstand, kroniske sykdommer og faste medisiner

Følgende spørsmål omhandler din generelle helsetilstand uavhengig av salmonellainfeksjonen

Har du noen kroniske sykdommer? *

- Ja
- Nei

Dette elementet vises dersom et av følgende alternativer er valgt på spørsmål «Har du noen kroniske sykdommer?»: Ja

Hvilke kroniske sykdommer har du? *

Du kan krysse av for flere alternativer:

- Kronisk diaré
- Leversykdom
- Immunsvekkelse
- Diabetes (sukkersyke)
- Magesår, gastritt, problemer med magesyren
- Mage-tarm-sykdom (inkludert Crohns og ulcerøs kolitt)
- Revmatisk sykdom (ledd- og bindevevssykdommer)
- Matallergi eller matintoleranse
- Annen kronisk sykdom

Dette elementet vises dersom et av følgende alternativer er valgt på spørsmål «Hvilke kroniske sykdommer har du?»: Matallergi eller matintoleranse

Hvilken matallergi eller matintoleranse har du? *

Dette elementet vises dersom et av følgende alternativer er valgt på spørsmål «Hvilke kroniske sykdommer har du?»: Annen kronisk sykdom

Hvilken annen kronisk sykdom har du? *

Er du operert i mage eller tarm noen gang? *

- Ja
- Nei

Dette elementet vises dersom et av følgende alternativer er valgt på spørsmål «Er du operert i mage eller tarm noen gang?»: Ja

Hva ble du operert for? *

- Brokk (lyskebrokk, navlebrokk eller annet)
- Fjernet galleblære eller hatt andre operative inngrep i galleblæren/gallegangene
- Blindtarmbetennelse
- Kreft i mage, tarm, bukspyttkjertel eller galleblære/galleganger
- Andre operasjon grunnet sykdomstilstander i mage-tarm systemet

Bruker du noen faste medisiner? *

- Ja
- Nei

Dette elementet vises dersom et av følgende alternativer er valgt på spørsmål «Bruker du noen faste medisiner?»: Ja

Hvilke faste medisiner bruker du? *

- Antibiotika (f.eks. penicillin)
- Syrenøytraliserende medisiner
- Insulin eller annen diabetesmedisin
- Kortisontabletter, steroider

- [ ] Midler mot magesår
- [ ] Andre immundempende medisiner
- [ ] Helsekost, kosttilskudd og vitaminer
- [ ] Naturmedisin
- [ ] Andre faste medisiner

Dette elementet vises dersom et av følgende alternativer er valgt på spørsmål «Hvilke faste medisiner bruker du?»: Andre faste medisiner

Hvilke andre faste medisiner? *

Hva slags kosthold har du? *

- [ ] Kosthold uten restriksjoner
- [ ] Vegetarianer
- [ ] Veganer
- [ ] Pescatarianer
- [ ] Glutenfri kost (som ved cøliaki)
- [ ] Laktosefri kost (intoleranse for melk og melkeprodukter)
- [ ] Annet kosthold/diett

Dette elementet vises dersom et av følgende alternativer er valgt på spørsmål «Hva slags kosthold har du?»: Annet kosthold/diett

Vennligst spesifiser kosthold/diett: *

Bruker du noen prebiotika eller probiotika i kostholdet ditt? *

Prebiotika er ufordøyelige karbohydrater (fiber) som stimulerer vekst av gunstige bakterier som finnes i tykktarmen.

Probiotika er kosttilskudd av levende mikroorganismer som kan forbedre bakteriebalansen i tarmen.

Eksempler er:

- Surmelk eller yoghurt med probiotika (f.eks. Activia, Actimelk, BioQ, Cultura og Biola)
- Pre - eller probiotika i dråpe-, tablett- eller pulverform

- ( ) Ja
- ( ) Nei

Dette elementet vises dersom et av følgende alternativer er valgt på spørsmål «Bruker du noen prebiotika eller probiotika i kostholdet ditt?»: Ja

Hvilke pre- eller probiotika bruker du? *

Du kan krysse av for flere alternativer:

- [ ] Surmelk eller yoghurt med probiotika (f. eks Activia, Actimelk, BioQ, Cultura og Biola)
- [ ] Dråper, tabletter eller pulver med pro- eller prebiotika
- [ ] Andre pro- eller prebiotika

Dette elementet vises dersom et av følgende alternativer er valgt på spørsmål «Hvilke pre- eller probiotika bruker du?»: Andre pro- eller prebiotika

Vennligst spesifiser hvilke pre- eller probiotika du bruker: *

Sideskift

Side 4

4. Reiseaktivitet før og etter salmonellainfeksjonen

Har du vært på reise utenfor Norge den siste uken før du fikk salmonellainfeksjonen? *

- ( ) Ja
- ( ) Nei

Dette elementet vises dersom et av følgende alternativer er valgt på spørsmål «Har du vært på reise utenfor Norge den siste uken før du fikk salmonellainfeksjonen?»: Ja

Hvilke(t) land reiste du til ? *

Dette elementet vises dersom et av følgende alternativer er valgt på spørsmål «Har du vært på reise utenfor Norge den siste uken før du fikk salmonellainfeksjonen?»: Ja

Når reiste du fra Norge (dato)? *

Hvis du er usikker, oppgi omtrentlig dato.

dd.mm.åååå

Dette elementet vises dersom et av følgende alternativer er valgt på spørsmål «Har du vært på reise utenfor Norge den siste uken før du fikk salmonellainfeksjonen?»: Ja

Når reiste du tilbake til Norge (dato)? *

Hvis du er usikker, oppgi omtrentlig dato.

dd.mm.åååå

Dette elementet vises dersom et av følgende alternativer er valgt på spørsmål «Har du vært på reise utenfor Norge den siste uken før du fikk salmonellainfeksjonen?»: Ja

Var du innlagt på sykehus under oppholdet ditt utenfor Norge? *

- Ja
- Nei

Har du vært på reise utenfor Norge etter at du ble frisk fra salmonellainfeksjonen? *

- Ja
- Nei
- Vet ikke

Dette elementet vises dersom et av følgende alternativer er valgt på spørsmål «Har du vært på reise utenfor Norge etter at du ble frisk fra salmonellainfeksjonen?»: Ja

Hvilke(t) land reiste du til? *

Dette elementet vises dersom et av følgende alternativer er valgt på spørsmål «Har du vært på reise utenfor Norge etter at du ble frisk fra salmonellainfeksjonen?»: Ja

Når reiste du fra Norge (dato)? *

Hvis du er usikker, oppgi omtrentlig dato.

dd.mm.åååå

Dette elementet vises dersom et av følgende alternativer er valgt på spørsmål «Har du vært på reise utenfor Norge etter at du ble frisk fra salmonellainfeksjonen?»: Ja

Når reiste du tilbake til Norge (dato)? *

Hvis du er usikker, oppgi omtrentlig dato.

dd.mm.åååå

Dette elementet vises dersom et av følgende alternativer er valgt på spørsmål «Har du vært på reise utenfor Norge etter at du ble frisk fra salmonellainfeksjonen?»: Ja

Var du innlagt på sykehus under oppholdet ditt utenfor Norge? *

- Ja
- Nei
- Vet ikke

Side 5

Sideskift

5. Kartlegging av kontakt med dyr

Bor du eller jobber du på en gård med husdyr? *

- Ja
- Nei

Dette elementet vises dersom et av følgende alternativer er valgt på spørsmål «Bor du eller jobber du på en gård med husdyr?»: Ja

Har du vårt på gården den siste uken før du ble syk? *

- Ja
- Nei
- Vet ikke

Dette elementet vises dersom et av følgende alternativer er valgt på spørsmål «Bor du eller jobber du på en gård med husdyr?»: Ja

Har du vårt på gården etter du ble frisk? *

- Ja
- Nei
- Vet ikke

Har du vært i kontakt med dyr i løpet av den siste uken før du ble syk med Salmonella? *

- Ja
- Nei
- Vet ikke

Dette elementet vises dersom et av følgende alternativer er valgt på spørsmål «Har du vært i kontakt med dyr i løpet av den siste uken før du ble syk med Salmonella?»: Ja

Har du hatt regelmessig kontakt med følgende dyr den siste uken før du ble syk? *

Du kan krysse avfor flere alternativer:

- Hund
- Katt
- Storfe (ku, kalv, okse)
- Fjørfe (høns, kylling, kalkun, ender, gjess)
- Gris
- Hest eller ponni
- Sau eller geit
- Burfugler (papegøye, undulat, kanarifugl og lignende)
- Kanin, marsvin, hamster, rotte, mus
- Reptiler, amfibier (slanger eller frosker for eksempel)
- Vilt (elg, hjort, rein, rådyr, hare, rev, bever, rype, skogsfugl)
- Ville dyr i fangenskap (dyrepark eller zoologisk hage)
- Andre dyr
- Nei, jeg har ikke hatt kontakt med dyr i uka før jeg ble syk

Dette elementet vises dersom et av følgende alternativer er valgt på spørsmål «Har du hatt regelmessig kontakt med følgende dyr den siste uken før du ble syk?»: Andre dyr

Hvilke andre dyr? *

Har du vært i kontakt med dyr i perioden etter at du ble frisk fra salmonellainfeksjonen? *

- Ja
- Nei
- Vet ikke

Dette elementet vises dersom et av følgende alternativer er valgt på spørsmål «Har du vært i kontakt med dyr i perioden etter at du ble frisk fra salmonellainfeksjonen?»: Ja

Har du hatt regelmessig kontakt med følgende dyr etter du ble frisk? *

Du kan krysse av flere alternativer:

- Hund

- ☐ Katt
- ☐ Storfe (ku, kalv, okse)
- ☐ Fjørfe (høns, kylling, kalkun, ender, gjess)
- ☐ Gris
- ☐ Hest eller ponni
- ☐ Sau eller geit
- ☐ Burfugler (papegøye, undulat, kanarifugl og lignende)
- ☐ Kanin, marsvin, hamster, rotte, mus
- ☐ Reptiler, amfibier (slanger eller frosk for eksempel)
- ☐ Vilt (elg, hjort, rein, rådyr, hare, rev, bever, rype, skogsfugl)
- ☐ Ville dyr i fangeskap (dyrepark eller zoologisk hage)
- ☐ Andre dyr
- ☐ Nei, jeg har ikke hatt regelmessig kontakt med dyr etter jeg ble frisk

Dette elementet vises dersom et av følgende alternativer er valgt på spørsmål «Har du hatt regelmessig kontakt med følgende dyr etter du ble frisk?»: Andre dyr

Hvilke(t) andre dyr? *

Har du kontakt med dyr gjennom arbeidet ditt? *

- ○ Ja
- ○ Nei

Dette elementet vises dersom et av følgende alternativer er valgt på spørsmål «Har du kontakt med dyr gjennom arbeidet ditt?»: Ja

Hva slags arbeid? *

Dette elementet vises dersom et av følgende alternativer er valgt på spørsmål «Har du kontakt med dyr gjennom arbeidet ditt?»: Ja

Hvilke dyr har du kontakt med? *

Side 6

Sideskift

6. Tilleggsopplysninger

Har du opplysninger som vi har ikke spurt etter men som du tror kan være relevante for studien?

Se nylige endringer i Nettskjema (v700_0rc1)
