# Supplementary material for: Risk factors associated with long-term shedding infections of non-typhoidal Salmonella in humans
Source: Eur J Clin Microbiol Infect Dis. 2025 May 27;44(9):2047–57. doi: 10.1007/s10096-025-05165-x (PMC12457537; doi:10.1007/s10096-025-05165-x)
Supplement: Supplementary file 2 — Supplementary Material 2 [file 10096_2025_5165_MOESM2_ESM.pdf]

Online Resource 2: Supplementary results

Article Title: Risk Factors Associated with Long-Term Shedding Infections of Non-Typhoidal *Salmonella* in Humans.

Authors: Andreas Rohringer[1,2*] (0000-0003-4351-7184), Lamprini Veneti[1*] (0000-0002-9117-5310), Anke Stüken[1], (0000-0002-7553-9524) Elburg van Boetzelaer (0000-0002-1168-8491)[3], Hilde M. Lund[1] (0009-0009-3290-4470), Zuzana Nordeng[1](0000-0002-2726-5952), Emily MacDonald[1], Umaer Naseer (0000-0002-1639-8397)[1]

1. Norwegian Institute of Public Health (NIPH), Oslo, Norway
2. European Programme for Public Health Microbiology (EUPHEM), European Centre for Disease Prevention and Control (ECDC), Stockholm, Sweden
3. European Programme for Field Epidemiology (EPIET), European Centre for Disease Prevention and Control (ECDC), Stockholm, Sweden

**Note: These authors contributed equally as first authors*
Corresponding author: Lamprini Veneti, Lamprini.Veneti@fhi.no

Journal name: European Journal of Clinical Microbiology & Infectious Diseases

1. Representativeness of study participants

We assessed the representativeness of study participants (included cases) by comparing their demographic characteristics—age group, sex, county of residence—with those of all salmonellosis cases notified to MSIS during the study period. Due to ethical constraints, we could not link the two datasets directly. Instead, we compared the distribution of characteristics by observing proportions across strata, without performing statistical tests. Overall, our participants represented salmonellosis cases well. We observed minor differences in age group distribution, with slightly fewer participants under 40 years and slightly more between 40-69 years of age. Additionally, female cases had a slightly higher participation rate.

Table S1: Distribution of *Salmonella* cases notified in 2019 Norway and included cases in the study (study participants) by age group, sex and county of residence.

	2019 Notified Cases (n=1,094)		Study Participants (n=255)	
**Age group**	n	%	n	%
0-9	127	11.61	20	7.66
10-19	103	9.41	14	5.36
20-29	170	15.54	26	9.96
30-39	126	11.52	17	6.51
40-49	146	13.35	48	18.39
50-59	175	16.00	55	21.06
60-69	155	14.17	55	21.06
70-79	67	6.12	23	8.81
80+	25	2.29	3	1.15
**Sex**				
Male	549	50.18	106	41.57
Female	545	49.81	149	58.43
**County of residence**				
Agder	48	4.39	12	4.71
Akershus	137	12.52	24	9.41
Buskerud	46	4.20	6	2.35
Finnmark	15	1.37	6	2.35
Innlandet	44	4.02	9	3.53
Møre og Romsdal	50	4.57	16	6.27
Nordland	37	3.38	10	3.92
Oslo	149	13.62	31	12.61
Rogaland	118	10.79	25	9.8
Telemark	30	2.74	8	3.14
Troms	37	3.38	11	4.31
Trøndelag	109	9.96	34	13.33
Vestfold	72	6.58	24	9.41
Vestland	146	13.35	27	10.59
Østfold	56	5.12	12	4.71

2. Potential risk factors associated with long-term shedding

In tables S2 and S3, we provide details on potential risk factors (sub-categories) presented in Tables 1 and 2 in the main article and additional ones that were assessed for the risk of long-term shedding (LTS) infections of non-typhoidal *Salmonella.* All questions assessed can be found in the study questionnaire (Online Resource 1).

Table S2: Univariable analysis assessing contact with animal and diet as potential risk factors for long-term shedding infections of non-typhoidal *Salmonella*.

		Total n (%)	Short-term shedding cases (n=193)		Long-term shedding cases (n=62)		Univariable analysis	
							OR	95% Confidence Interval
			n	%	n	%		
**Animal contact**								
**Live or work in a farm with livestock**	No	241 (95%)	185	77%	56	23%	Ref	Ref
	Yes	10 (3.9%)	5	50%	5	50%	3.30	0.92-11.82
	Missing#	4 (1.6%)	3	75%	1	25%		
**Been in a farm the week before having *Salmonella* infection detected**	No	3 (1.1%)	1	33%	2	67%	Ref	Ref
	Yes	6 (2.4%)	4	67%	2	33%	0.25	0.01-4.73
	Missing#	246 (97%)	188	76%	58	24%		
**Animal contact the week before having *Salmonella* infection detected**	No	158 (62%)	120	76%	38	24%	Ref	Ref
	Yes	82 (32%)	62	76%	20	24%	1.02	0.55- 1.90
	Missing#	15 (6%)	11	73%	4	27%		
**Animal contact after recovering from *Salmonella* infection**	No	107 (42%)	79	74%	28	26%	Ref	Ref
	Yes	135 (53%)	105	78%	30	22%	0.81	0.45-1.46
	Missing#	13 (5%)	9	69%	4	31%		
**Work-related animal contact**	No	245 (96%)	185	76%	60	24%	Ref	Ref
	Yes	7 (3%)	6	86%	1	14%	0.51	0.0-4.35
	Missing#	3 (1%)	2	67%	1	33%		
**Diet**								
No restrictions	No	27 (11%)	18	67%	9	33%	Ref	Ref
	Yes	228 (89%)	175	77%	53	23%	0.61	0.26-1.43
Vegetarian	No	255 (100%)	193	76%	62	24%	Ref	Ref
	Yes	0 (0%)	0	0%	0	0%	- †	- †
Vegan	No	255 (100%)	193	76%	62	24%	Ref	Ref
	Yes	0 (0%)	0	0%	0	0%	- †	- †
Pescetarian	No	255 (100%)	193	76%	62	24%	Ref	Ref
	Yes	0 (0%)	0	0%	0	0%	- †	- †
Gluten free	No	250 (98%)	190	76%	60	24%	Ref	Ref
	Yes	5 (2%)	3	60%	2	40%	2.11	0.35-12.9
Lactose free	No	247 (97%)	190	77%	57	23%	Ref	Ref
	Yes	8 (3%)	3	38%	5	63%	5.56*	1.29-23.9

Other	No	238 (93%)	182	76%	56	24%	Ref	Ref
	Yes	17 (7%)	11	65%	6	35%	1.77	0.63-5.01

** P-value < 0.05*

# Missing values were not included in univariable analysis

† : Estimates could not be provided as no participants were exposed to the assessed potential risk factor

Table S3: Univariable analysis assessing medication use, and surgery history as potential risk factors for long-term shedding infections of non-typhoidal *Salmonella*.

		Total n (%)	Short-term shedding cases (n=193) n	%	Long-term shedding cases (n=62) n	%	Univariable analysis OR	95% Confidence Interval
**Use of regular medication (Not salmonellosis related, including the following options)**	No	115 (45%)	95	83%	20	17%	Ref	Ref
	Yes	138 (54%)	97	70%	41	30%	2.01*	1.10-3.68
	Missing#	2 (0.8%)	1	50%	1	50%		
Antibiotics	No	251 (98%)	189	75%	62	25%	Ref	Ref
	Yes	4 (2%)	4	100%	0	0%	0.77	0.08-7.06
Antacids	No	222 (87%)	168	76%	54	24%	Ref	Ref
	Yes	33 (13%)	25	76%	8	24%	0.99	0.42-2.34
Insulin	No	248 (97%)	187	75%	61	25%	Ref	Ref
	Yes	7 (3%)	6	85%	1	14%	0.51	0.06-4.33
Corticosteroids	No	251 (98%)	192	76%	59	24%	Ref	Ref
	Yes	4 (1.6%)	1	25%	3	75%	9.76*	1.00-95.62
Ulcer medication	No	255 (100%)	193	76%	62	24%	Ref	Ref
	Yes	0 (0%)	0	0%	0	0%	- †	-†
Immunosuppressives	No	249 (98%)	190	76%	59	24%	Ref	Ref
	Yes	6 (2%)	3	50%	3	50%	3.22	0.63-16.4
Food supplements	No	231 (91%)	176	76%	55	24%	Ref	Ref
	Yes	24 (9%)	17	71%	7	29%	1.32	0.52-3.34
Nature	No	255 (100%)	193	76%	62	24%	Ref	Ref
	Yes	0 (0%)	0	0%	0	0%	- †	- †
Other	No	139 (55%)	115	83%	24	17%	Ref	Ref
	Yes	116 (45%)	78	67%	38	33%	2.33*	1.30-4.20
**Abdominal surgery prior to infection**	No	209 (82%)	161	77%	48	23%	Ref	Ref
	Yes	45 (18%)	31	69%	14	31%	1.51	0.75-3.08
	Missing#	1 (0.4%)	1	100%	0	0%		
Hernia	No	247 (97%)	188	76%	59	24%	Ref	Ref
	Yes	8 (3.1%)	5	63%	3	37%	1.91	0.44-8.24
Gallbladder	No	250 (98%)	191	76%	59	24%	Ref	Ref
	Yes	5 (2.0%)	2	40%	3	60%	4.86	0.79-29.8
Appendix	No	235 (92%)	179	76%	56	24%	Ref	Ref

	Yes	20 (7.8%)	14	70%	6	30%	1.37	0.50-3.73
Cancer	No	255 (100%)	193	76%	62	24%	Ref	Ref
	Yes	0 (0%)	0	0 (0%)	0	0 (0%)	- †	- †
Other	No	240 (94%)	181	75%	59	25%	Ref	Ref
	Yes	15 (5.9%)	12	80%	3	20%	0.77	0.21-2.81

*: P-value < 0.05

#: Missing values were not included in univariable analysis

† : Estimates could not be provided as no participants were exposed to the assessed potential risk factor

3. Prolonged Symptoms

Approximately five weeks after the initial sample, 35 participants (14%) reported experiencing symptoms (Table S4). Duration for the reported symptoms could not be determined based on our questionnaire. No difference was observed in the distribution of prolonged symptoms between participants with STS and LTS (chi square p-values > 0.05).

Table S4: Distribution of reported symptoms present five weeks after the initial sample by short-term shedder or and long-term shedder phenotypes of non-typhoidal *Salmonella*.

	**Present symptom five weeks after the initial sample**					
	**Short-term shedding cases (n=193)**		**Long-term shedding cases (n=62)**		**Total (n=255)**	
**Symptom**	n	%	n	%	n	%
Nausea	1	0.5%	1	1.6%	2	0.8%
Abdominal pain	9	4.7%	6	9.9%	15	5.9%
Blood in stool	4	2.1%	1	1.6%	5	2.0%
Vomiting	0	0%	0	0%	0	0%
Joint pain	2	1.0%	1	1.6%	3	1.2%
Mucus in stool	7	3.6%	5	8.1%	12	4.7%
Diarrhea	6	3.1%	3	4.8%	9	3.5%
Fever	0	0%	0	0%	0	0%
Other symptoms	12	6.2%	4	6.5%	16	6.3%
**Asymptomatic**	168	87%	52	84%	220	86%

4. Hospitalisation

Table S5: Hospitalisation as reported from study participants by serotype and by short-term shedder and long-term shedder phenotypes of non-typhoidal *Salmonella*.

Serotype	Total cases assessed (n=255)		Short-term shedding cases (n=193)		Long-term shedding cases (n=62)	
	Total cases	Hospitalised cases	Total cases	Hospitalised cases	Total cases	Hospitalised cases
Enteritidis	107 (42%)	34 (32%)	84 (79%)	24 (29%)	23 (21%)	10 (43%)
Typhimurium	27 (11%)	11 (41%)	22 (81%)	9 (41%)	5 (19%)	2 (40%)
Agbeni	22 (9%)	5 (23%)	12 (55%)	2 (17%)	10 (45%)	3 (30%)
Monophasic Typhimurium	16 (6%)	6 (38%)	12 (75%)	5 (42%)	4 (25%)	1 (25%)
Stanley	14 (5%)	5 (36%)	11 (79%)	3 (27%)	3 (21%)	2 (67%)
Newport	9 (4%)	2 (22%)	8 (89%)	2 (25%)	1 (11%)	0 (0%)
Coeln	7 (3%)	1 (14%)	5 (71%)	0 (0%)	2 (29%)	1 (50%)
Other	53 (21%)	16 (30%)	39 (74%)	12 (31%)	14 (26%)	4 (29%)
**Total**	255 (100%)	80 (31%)	193 (76%)	57 (30%)	62 (24%)	23 (37%)
